# The Strengths and Difficulties Questionnaire is a usable way to address mental health at well-child visits in general practice - a qualitative study of feasibility

**DOI:** 10.1186/s12875-020-01156-3

**Published:** 2020-07-01

**Authors:** Julie Ravneberg Stokholm, Kirsten Lykke

**Affiliations:** Department of Public Health, The Research Unit for General Practice and Section of General Practice, Faculty of Health Sciences, University of Copenhagen, Denmark, Copenhagen, Denmark

**Keywords:** Strength and difficulties questionnaire / SDQ, Preschool children, Mental health, Psychopathology, general practitioner/ physicians, primary care, Questionnaire, Preventive health service, Well-child visit, Routine childcare visits

## Abstract

**Background:**

Mental health problems is frequent among children and psychopathology in early childhood seems to predict mental disorders in adulthood. All Danish children are offered seven free well-child visits at their General Practitioner (GP) during their first 5 years of life. GPs have a unique position to address mental health problems at the well-child visits, but they lack a systematic approach when assessing children’s mental health. The purpose of this study was to investigate if the Strengths and Difficulties Questionnaire (SDQ) is a usable way to address preschool children’s mental health in general practice.

**Methods:**

A qualitative study of feasibility. Parents completed an online version of the SDQ at home. At the well-child visit, the GP used the SDQ results as a basis for a talk about the child’s mental health. Afterwards the author JS conducted semistructured interviews with both the parent and the GP over the phone. The interviews were descriptively analyzed using the Framework Approach.

**Results:**

Five primary care centres with 22 general practitioners in both Copenhagen and Region Zealand participated. Twenty four parents completed the SDQ and were interviewed. Participating parents and GPs agreed, that the SDQ introduced mental health as a natural and important part of the well-child visit. Online access had clear advantages: time for reflection at home and preparation, plus a clear result summary for the GP. Some of the GPs were worried that the questionnaire would be too time consuming, and might compromise the individualistic style of general practice. Some parents were worried if children with minor problems would be diagnosed.

**Conclusions:**

The online SDQ was well-accepted and feasible in daily practice. Implementing the SDQ into the well-child visit could strengthen the focus on the child’s mental health. However, before the SDQ can be generally implemented a guideline on how to utilize it in the well-child visit is needed, as well as studies of efficacy in this setting.

**Trial registration:**

Not relevant.

## Background

Mental Health (MH) problems are believed to constitute more than 50% of the total burden of disease for Danish children and young people [1] and a similar picture is seen worldwide [2]. A systematic review from 2015 found the worldwide-pooled prevalence of mental disorders affecting children and adolescents to be 13,5% [3]. Early identification and intervention can relieve children of current symptoms and alter a trajectory of mental illness [4]. Early interventions also have the advantage of helping before the child develops a negative self-image and before complicating factors emerge, such as comorbid psychiatric disorders, academic failure, and poor social relationships [5, 6]. Moreover, researchers have argued for early intervention, as the young brain is likely to be more plastic and more susceptible to lasting modifications [5].

All Danish children are offered seven free well-child visits at their General Practitioner (GP) during their first 5 years of life. According to guidance from the Danish Health and Medicines Authority, the well-child visit should be an assessment of the child’s “physical, psychological and social development and well-being” [7]. In this guidance, the GPs are encouraged to talk with the parents about the psycho-social health, but there is no specific guidance on how to implement this dialogue or recommendations of standardized methods. The GPs have a unique position to assess the child’s well-being because: 1) they have continuous contact with the families, 2) the parents often have confidence in the GP and 3) physical symptoms and psychosocial symptoms often occur together in small children [8]. Unfortunately, GPs seem to lack systematism and a theoretical framework to approach mental health problems in children [9]. They report a lack of knowledge to detect and manage mental health problems in children [10–12]. They also seem to lack a method to involve parents in their specific observations and concerns about a child [13].

All put together, the GPs seems to be lacking an evidence-based method for assessing mental health, which is central to ensure the quality of the well-child visits.

The GPs can observe the child and its interaction with the parent, but they are also reliant on the parents’ information to find children with mental health problems. Sayal found that GPs’ identification of children who had mental health problems increased from 26 to 88% *if* the parent expressed concern at the consultation. But only a third of parents who had concerns, expressed these during the consultation [14]. The same author identified in another study the factors that influence parents’ decision to seek help in primary care with concerns for their child’s mental health. Factors that increased help seeking were: knowing that the GP also deals with mental health problems, trust in the GP and the health care system and time set aside per visit. As barriers of help seeking, the parents named concern of labelling the child with a diagnosis or themselves as bad parents [15].

Questionnaires have been suggested as a means to make mental health a natural part of well-child visits, and to identify children at elevated risk [8, 16, 17]. For this purpose, the Strength and Difficulties Questionnaire (SDQ) might be a useful tool [8, 18]. The SDQ is a brief instrument developed in England by Robert Goodman in 1997. It is available in a variety of versions for different age groups and types of respondents (parents, teachers or the child itself) and is translated into over 70 languages. It consists of 25 questions, which are divided between five scales with five items each: 1) emotional symptoms, 2) conduct problems, 3) hyperactivity/inattention, 4) peer relationship problems and 5) prosocial behavior. The first four scales added together generate the Total Difficulty Score. The respondents answer the 25 questions based on the child’s behavior over the last 6 months. Then they are asked to evaluate if the child overall has problems in relation to emotions, concentration, behavior or being able to get on with other people. If they reply “yes”, they are asked further about chronicity, distress, social impairment and burden to others – thus giving information about the total impact on daily life [19, 20]. The SDQ is not a diagnostic tool, but it can point out which areas the child has difficulties in and what impact the difficulties have on daily life. The SDQ is widely used internationally, e.g. in New Zealand, where the SDQ is a part of the “B4 School Check” - a nationwide health check of preschool children [21]. The SDQ has shown to be well functioning and with sound psychometric properties in a Danish setting as well [22].

Before introducing a questionnaire into the well-child visits, it must be evaluated for its feasibility in daily practice. The aim of the current study is to investigate the feasibility of the online SDQ in general practice, and the questionnaire’s capability to introduce mental health as a natural part of the well-child visits.

## Method

### Aim

The aim of the current study is to investigate the feasibility of the online SDQ in general practice, and the questionnaire’s capability to introduce mental health as a natural part of the well-child visits.

### Design

This is a qualitative study of the feasibility of the Strength and Difficulties Questionnaire (SDQ) in an online version. We introduced the SDQ in the well-child visits as a basis for a talk about the child’s mental health. Outcome data consisted of a descriptive analysis of semi-structured interviews with parents and GPs. Julie Ravneberg Stokholm (JS) and Kirsten Lykke (KL) composed the interview guides (Table 1).

**Table 1:** Interview guides

**Interview with General Practitioners (GP)** - How many years have you been working as a GP? - Did you know the family/the child already? - What is your perception of talking about preschool children’s mental health at the well-child visits up to now? - Did you find the questionnaire easy to use? - How do you think the questionnaire affects the time spent on assessing the child’s psycho-social health? - How did you and the parent use the questionnaire in your conversation? - Did you experience that the SDQ affected the contents of the well-child visits? - Do you think that completing a questionnaire from home affects the parents’ approach to the well-child visits? - What would you think if a questionnaire like this was to be implemented in the well-child visits?	
**Interview with parents** - Did you find the questionnaire easy to complete? - Completing the questionnaire, did it bring anything to mind or did it change how you think (Name) is doing? - How did you and the doctor use the questionnaire in your conversation? - Do you think that completing the questionnaire at home influenced the conversation with the doctor?	

### Setting

The GPs were recruited through personal networks and through an internal homepage for GPs in Copenhagen. Eleven GPs in five primary care centres in Copenhagen and in the region of Zealand participated. The GPs were purposedly selected in order to involve respondents from both rural and urban areas. It was not systematically registered how many declined or did not react to the invitation. We chose the sample size based on what we from experience estimated would be suitable as well as feasible. The included child health examinations took place in January and February 2016. All well-child visits booked for children at age 4–5 in the trial period were included, thus the sampling of the families was consecutive. Exclusion criteria was insufficient Danish language by the parent, but there were no recorded exclusion on this background. It was not registered if the parents declined to participate. Tables 2 and 3 present the characteristics of the participating GPs and families.

**Table 2:** Basic information on the involved general practitioners

	Female/ Male	Location	Time allotted per well-child visit	Number of well-child visit included in the study
GP1	F	Countryside	15 min	3
GP2	M	Countryside	15 min	1
GP3	F	Countryside	15 min	2
GP4	M	Countryside	15 min	1
GP5	M	Countryside	15 min	2
GP6	M	Countryside	30 min	4
GP7	F	Countryside	30 min	1
GP8	F	Copenhagen	30 min	1
GP9	M	Copenhagen	30 min	4
GP10	F	Copenhagen	30 min	4
GP11	F	Copenhagen	30 min	2

**Table 3:** Basic information on the families, who completed the SDQ and were interviewed

Family number	Gender of the child	Place of residence	Who completed the questionnaire?	Who was interviewed?
1	Girl	Countryside	Mother	Mother
2 (twins)	Boy + Girl	Countryside	Mother	Mother
3	Girl	Countryside	Mother	Mother
4	Boy	Countryside	Mother	Mother
5	Girl	Countryside	Mother	Mother
6	Girl	Countryside	Mother	Mother
7	Girl	Copenhagen	Mother and father	Mother
8	Boy	Copenhagen	Mother	Mother
9	Boy	Copenhagen	Mother	Mother
10	Girl	Copenhagen	Mother	Mother
11	Girl	Countryside	Mother	Mother
12	Girl	Countryside	Mother	Mother
13	Boy	Copenhagen	Mother and father	Father
14	Girl	Copenhagen	Mother	Mother
15	Girl	Copenhagen	Mother	Father
16	Boy	Copenhagen	Mother	Mother
17	Boy	Countryside	Mother	Mother
18	Boy	Countryside	Mother	Mother
19	Boy	Countryside	Mother	Mother
20	Girl	Copenhagen	Mother	Mother
21	Girl	Countryside	Mother	Mother
22	Boy	Countryside	Mother	Mother
23	Boy	Copenhagen	Mother and father	Mother
24	Girl	Countryside	Mother	Mother
**Total of 24 interviews**	**Girls: 14** **Boys: 11**	**Countryside: 14** **Copenhagen: 10**	**Mother: 21** **Father: 0** **Both: 3**	**Mother: 22** **Father: 2**

### Description of process

JS visited all the primary practice centres and gave a short introduction to the GPs on how to use the online SDQ, as they did not have prior experience with it. JS had subsequently continuous contact with each primary practice centre by telephone, and received information for each new booking of a well-child visit. JS called the parents and introduced herself as a medical student, and asked if they would like to participate in the study. If they consented, JS send them an e-mail with additional information and a personal login for the webpage www.besvarsdq.dk. It was usually the mother’s telephone number which was given from the clinic, and thus the mother that JS called. The parents were informed that both parents could answer the questionnaire, but this was optional.

On the day of the well-child visit, the GP logged on to www.besvarsdq.dk and checked the child’s SDQ results. The answers to the questions were divided into the five scales: 1) emotional symptoms, 2) conduct problems, 3) hyperactivity/inattention, 4) peer relationship problems and 5) prosocial behavior. Each scale was shown with the child’s score, and the Total Difficulty Score was stated. The child’s scores were automatically classified as” normal”,” slightly raised”, “high” or “very high” and marked green, yellow, orange or red, respectively. To have a “very high” score meant that more than 95% of children would have a lower score. The corresponding numbers for “high” was 90% and for “slightly raised” was 80%. The cut-off values used for this interpretation were based on an English population, which have shown to be close to Danish conditions [17, 18]. In addition to the scores, the GPs could see if the parent reported that the child overall had problems, and if so, how much it had *impact* on daily living.

The parents were interviewed within about 1 week after the well-child visit. In order to minimize time consumption needed for the GPs to participate in the study, they were only interviewed once they had completed all their well-child visits. JS performed all interviews and they were done by telephone and audio recorded.

### Data analysis

**Fig. 1:**
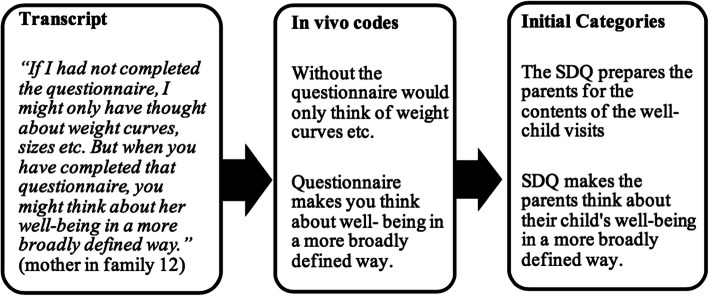
Example of the analysis using the framework approach. From the transcript in vivo *codes* were extracted using the informant’s own words. JS then rephrased the in vivo *codes* to *initial categories.* Similar categories were eventually brought together to form themes (this step is not shown in the figure)

We chose the “framework approach method” for the analysis. The method enables a structured step-by-step analysis that make the process transparent and illustrates the linkage between the stages of the analysis [23]. JS performed, audio recorded and transcribed verbatim all interviews. JS then identified key statements and gave them a headline (in vivo *code*) using the participants’ own words. From these in vivo codes, *initial categories* were developed (Fig. 1). JS and KL continuously discussed the step-by-step analysis of the first dozen interviews and identified four main themes that the initial categories could be divided into. The four main themes identified were: 1) how mental health is usually handled in well-child visit, 2) how the SDQ prepared both GPs and parents for the well-child visit, 3) what influence the SDQ had on the well-child visit and 4) opinions on implementing the SDQ in the well-child visits. JS wrote descriptive accounts for each theme by summarizing and synthesizing all the related initial categories.

## Results

Thirty-eight parents agreed to participate in the study. However, seven did not complete the SDQ. Of the 31 completed SDQs, the GPs did not use it in six cases, thus the SDQ was completed *and* used in 25 cases. Reasons for not completing or not using the SDQ were technical problems or forgetfulness. From these 25 cases, interviews with *both* a parent and the GP were achieved in 21 cases. All parents who had completed the SDQ were interviewed if possible, also those who did not get to use it at the well-child visit, thus a total of 24 interviews with parents were made. Two of the children were twins, why they only resulted in one interview. In total, 11 GPs and 24 parents were interviewed. Table 2 present characteristics of the GPs and Table 3 presents the characteristics of the involved families. As Table 3 shows, it was generally the mothers who completed the questionnaires and were interviewed.

As mentioned, the analysis of the interviews revealed four main themes, which will be presented in the following. The four main themes were: 1) how mental health is usually handled in well-child visit, 2) how the SDQ prepared both GPs and parents for the well-child visit, 3) what influence the SDQ had on the well-child visit and 4) opinions on implementing the SDQ in the well-child visits.

### The usual well-child visit - expectations and experiences

In general, parents said that they did not normally associate the well-child visit with talking about mental health problems. The parents often forgot, or did not think of, mentioning their thoughts and worries. The GPs expressed very different methods to evaluate the child’s mental health. Some GPs said they did not bring mental health up unless the parents did, or if they knew of problems beforehand. Others had quite established routines with standard questions and observations of the child’s behavior and contact with the parent. Some GPs emphasized that they hoped the parents would bring up concerns if they had any. The GPs often encouraged the parents to do so, by using open questions like” How are things in the kindergarten?” This was a way to address the subject without being judging towards the parent. One GP noticed that it was difficult to talk about mental health problems in vulnerable families who often tried to hide problems, and thus problems were easily neglected. In general, the GPs pointed out the importance of having a gut feeling” if something is wrong” and accentuated the importance of knowing the families and children through many years. Nonetheless, one GP commented:” *many of these things - you imagine that you know because you know the family already, but of course you don’t know everything after all”* (GP5).

### The SDQ as a preparation for the well-child visit

Parents and GPs experienced that the SDQ was a good preparation for the well-child visit. The parents emphasized the advantage of completing the SDQ at home, where they had time and peace to think. As one mother said:*“you aren’t very present when you sit at the doctor’s … there, you might be like ‘I can’t remember’”* (Family 3). Another mother felt that completing the questionnaire helped to avoid ready-made answers at the GP’s. The questionnaire had made many parents reflect on their child, but the majority did not think that completing the SDQ had changed their view of their child. In general, GPs appreciated that the SDQ informed the parents that mental health was a part of the well-child visit. One GP said,” *Instead of sitting at the doctor’s and saying something out of consideration for the doctor […] You [the parent] can sit at home and write down how things really are, and this can serve as the basis for the conversation”* (GP10). The benefits of preparation were illustrated in a case with a child who had high scores on the SDQ. Both the father and the GP experienced that the SDQ helped them to have a good conversation on this difficult topic. The father said, *“I think that the reluctance about what you can and will talk about in such a conversation was sort of removed, and you entered a more open dialogue. It [the SDQ] paved the way,”* (Family 13). The GP said, *“Sometimes it is quite difficult, you have a child who clearly isn’t age-appropriate, and then you have to find out*” *Where are the parents in this?” […] And here I think that this questionnaire can prepare the parents for some of the questions that I am going to ask them.”* (GP11).

The parents found that the SDQ was easy and quick to complete on the homepage. In general, GPs found that the SDQ provided a quick and easy overview. Some, especially those who only had one well-child visit with SDQ, thought it was too time-consuming to check the answers on the homepage. Two GPs thought that the SDQ was too extensive.

### Well-child visits with the use of SDQs

Parents and GPs thought that the SDQ had a positive influence on the well-child visit. The parents believed that the SDQ had made the GP more aware of mental health, and appreciated the questionnaire as a natural starting point for the conversation. The GPs experienced that the questionnaire had given them a more detailed insight into the well-being of the child, and had inspired them to address the subject in new ways. *“After the questionnaire I think that it has become much more nuanced, and I have become more attentive to what you can enquire into, and I have become more inspired as to what kind of questions I can ask”* (GP10). A couple of GPs specifically emphasized the usefulness of the” impact on everyday life” question, as it made the parent decide if they thought the symptoms were a problem or not.

In general, GPs believed that the SDQ could be helpful in identifying children in the “grey area”: those who do have problems, but not severe enough to be recognized in the present practice. On the other hand they were worried if some parents might be inclined to report small and insignificant problems in the questionnaire. One GP had a consultation were both parents had completed the SDQ but with different answers. The child seemed quite normal at the consultation and the GP experienced that this discrepancy was a good opportunity to talk about what is normal to expect from a 4-year-old, and to reassure the parents that their child appeared quite normal.

A recurring GP comment was that, as the SDQ comprised more than the GPs would normally ask about in their well-child visit, it also took up more time. The GPs with the least time set aside for well-child visit were more likely to find the SDQ too time-consuming.

### Opinions on implementing the SDQ in the well-child visits

Parents and GPs were generally positive towards implementing an online SDQ in the well-child visit. The parents were not asked directly about their opinion on implementation, but several commented on it on their own initiative. They thought that the SDQ would get both parents and GPs to talk about mental health and give structure to the conversation. Some parents were worried if the answers in the SDQ would be used uncritically, and children with minor problems would be diagnosed. The GPs were generally positive towards implementing an online SDQ in the well-child visit. They experienced that the SDQ gave them a more detailed view of the child’s well-being and a good starting point for the conversation with the parents. Some GPs had concerns that the SDQ would cost extra time and that too many forms and questionnaires could be a threat to the individualistic style in general practice:” *When you have been in the profession for many years, it is good to be shaken a bit, and that I think is fine. But in general, I like that you are allowed to be individualistic in your consultations.”* (GP2). One GP suggested that the GP could use the SDQ on a case-by-case basis as a solution.

## Discussion

In this study, the online SDQ was an easy and accessible tool for both parents and GPs. The SDQ introduced mental health as a natural part of the well-child visit, and it helped the GP to obtain a more detailed impression of the child’s well-being. The questionnaire being online had several advantages: 1) time and peace for reflection at home, 2) preparing both parents and GPs and 3) providing a quick overview of the SDQ results for the GP. Objections from the GPs were few and mainly focused on time consumption and protection of the individualistic style as general practitioners. The parents were generally very positive towards the online SDQ, but a couple of parents were worried if children with minor problems would be diagnosed.

### Comparison of findings with literature

GPs have requested tools for addressing the children’s mental health in Danish, English and Canadian contexts [10–12]. We introduced the SDQ to the GPs and parents as a communication tool to assess mental health in the well-child visit. When the SDQ is used in this way, to support the conversation with the parents, it resembles the use of Patient Reported Outcome Measures (PROM). PROM are questionnaires completed by the patient before a consultation, and tailored to address symptoms of a specific condition [24]. The advantages are: *preparing* the patient for conversation with the clinician, *engaging* patients in their own treatment, *improved communication* especially concerning sensitive questions, and a more *needs-based* approach to treatment [25]. Thus the advantages of PROMs are very similar to those found in our study using the SDQ in mental health assessment. Participants emphasized especially the preparation, as many of the parents were not aware that mental health was a part of the health check. This inadequate knowledge of the well-child visits was also noticed in the national evaluation of the Danish well-child visits [12]. With the completion of the online SDQ, the parents had reflected on their child at home, and found it easier to engage in a constructive dialogue with the GP. Thus, one could argue that the SDQ increases the parents’ health literacy, as they became aware of the intentions of the well-child visits and thus were better prepared to engage in it.

The participants emphasized several advantages of the questionnaire being online. If the online SDQ should be implemented in well-child visits, we must consider response rates on online questionnaires. In a Danish study, over 3000 parents were invited to complete a questionnaire on their children’s health for research purposes. The response rates between a paper- and web-based version of the questionnaire were compared and found to be similar but overall not very high [26]. However, if the online SDQ were implemented as a preparation for the well-child visits, the parents would have a personal interest in completing the questionnaire, instead of doing it “for the sake of science”, which might increase the response rate. This possibility was also discussed in the above-mentioned paper, as a way to yield valuable epidemiological data [26]. In many Danish municipalities, an online version of the SDQ (in combination with other questionnaires on children’s health) is already being used in schools, daycare centres etc. This is done through a multidisciplinary platform developed in a collaboration between scientists from Aarhus University and the municipalities [27]. All put together, an online version of SDQ could potentially work as a dialogue tool between parents, doctors, teachers etc. and concurrent produce useful data for further research.

In our study, a couple of parents, but none of the GPs, mentioned a concern of over-diagnosing. This is a relevant concern, as a psychiatric diagnosis is associated with stigma and might affect the child’s self-perception. Over-diagnosing could also lead to a rise in unnecessary referrals to child psychiatry.

On the other hand, improved communication about mental health gives GPs more insight into the child’s well-being, and thus qualifies their assessment. If we should implement the SDQ it would be important to communicate to both parents and GPs, that the SDQ is not a diagnostic tool, and that a diagnosis is given by a doctor, not a questionnaire. Also the GPs should receive instructions or training in how to handle the SDQ results, including information on positive/negative predictive value and the need of clarifying questions and recommendations for further action [28].

Time consumption is central when assessing the feasibility of the SDQ. A conversation about mental health can potentially be very time consuming, as the subject is delicate and complex. Some of the GPs in our study found that the SDQ was too extensive and therefore took up too much time. On the other hand, one could argue that the SDQ increases the efficacy of the visit, as the GP can address the areas of concerns directly, instead of beginning the consultation with asking a battery of questions. The evaluation on time consumption using the SDQ depends on the available time frame and the usual way the GP addresses mental health. In our study, the time frame for the well-child visits were either 15 or 30 min (Table 2) which naturally affects the GPs evaluation on time consumption. If the GP usually ask a non-specific question like “how is kindergarten?”, the answer will most likely be “good”, and the subject matter is thus quickly done with. The SDQ naturally invites to a more thorough talk on mental health than a single question.

One GP proposed that the individual GP could use the SDQ only in situations of special concern, thus minimalizing the time consumption and preserving the individualistic style in general practice. However, research has shown that parents find it acceptable to answer questions about mental health *because* they know that all parents receive the same questionnaires [28, 29]. Moreover, studies report that doctors’ sensitiveness towards children’s mental health problems is low, and parents often do not disclose concerns on this subject [14]. Additionally, if the GPs only used the SDQ occasionally, they would miss the positive side effects of the SDQ: preparation, systematism and a good starting point for the conversation. As mentioned in the background section, mental health (MH) problems are believed to constitute more than 50% of the total burden of disease for Danish children and young people [1]. One could argue that the priority of mental health in well-child visits should reflect this scale of disease burden.

The SDQ has been available in Denmark for several years and is approved as a psychometric tool under the general agreement between the GPs and the government. The official guidelines for the well-child visit does however not refer to the SDQ. The SDQ is a valid tool for assessing children’s mental health [22] and this study indicates that an online SDQ is feasible in daily practice. The SDQ was a well-accepted, easy and systematic way for the GP to ask:*“How are things?”*

### Limitations

The GPs who agreed to participate in the study might have a special interest in children and/or psychiatry and a more positive attitude towards the questionnaire than most. On the other hand, the GPs had very different thoughts and approaches on mental health in the well-child visit, which could speak for a diverse composition of GPs. This is backed up by the characteristics of the participating GPs, as there was an equal gender distribution and GP practices from both the capital and countryside.

It is a limitation that each GP only had few well-child visits using the SDQ and therefore had little experience to base their evaluation on. We decided to interview all the participating GPs, also those who had only one well-child visit with the SDQ, because we wanted to increase the number of informants and thereby the versatility of the experiences.

The parents who participated might represent resourceful parents, and it was not systematically registered if a parent declined to participate. According to the general practice secretaries, invitation to participate was generally accepted, and likewise, none of the parents who were asked by JS declined to participate. JS had continuous contact with GPs, secretaries and parents which contributed to a relatively low degree of drop-outs.

As Table 3 clearly shows, it was typically the mothers who completed the questionnaire and were interviewed. Additionally, statistics show that between 20 and 30% do not attend well-child visits for 4- and 5-year-old children. Non-attendance is associated with socio economic factors like the mother’s employment and education etc., but also if the child has older siblings [12, 30]. Thus one could question if the results of our study are transferable to the families and fathers who do not attend. The purpose of this study was however to investigate the feasibility of the SDQ in the existing context of well-child visits, and is thus subject to the same limitations.

## Conclusions

In this study, an online SDQ was well-accepted and feasible in daily practice. Implementing the SDQ into the well-child visit could strengthen the focus on the child’s mental health. However, before the SDQ can be generally implemented, a guideline on how to utilize it in the well-child visit is needed, as well as studies of efficacy in this setting. SDQ could be introduced into the well-child visit in one region followed by research on efficacy and acceptability.

When increasing the focus on mental health problems, the GPs will probably become aware of more children in the “grey zone”, meaning those who have problems, but not enough to meet criteria for referral. This leaves the GPs with a need for methods to deal with mental health in practice. Thus, the development of practical interventions is a crucial next step. As an example, these interventions could be online family therapy, further education of GPs, municipal activities or contact with the child’s institution or school. Additionally the GPs would also find more children who need referral to child psychiatry, and this raises another concern: does the child psychiatry have the capacity to handle a growing numbers of children with mental health problem.

## Data Availability

The datasets used and analysed during the current study are available from the corresponding author on reasonable request.

## Abbreviations

MH: Mental Health
GP: General Practitioner
SDQ: Strengths and Difficulties Questionnaire
PROM: Patient Reported Outcome Measures
JS: Julie Ravneberg Stokholm (author)
KL: Kirsten Lykke (author)
